# Experiments and CFD simulation of an air-conditioned tractor cabin for thermal comfort of tractor operators in Pakistan

**DOI:** 10.1016/j.heliyon.2023.e23038

**Published:** 2023-11-29

**Authors:** Mahmood Riaz, Muhammad Hamid Mahmood, Muhammad Nauman Ashraf, Muhammad Sultan, Uzair Sajjad, Khalid Hamid, Muhammad Farooq, Faming Wang

**Affiliations:** a Department of Agricultural Engineering, Faculty of Agricultural Sciences & Technology, Bahauddin Zakariya University, Multan, 60800, Pakistan; b Department of Energy and Refrigerating Air-Conditioning Engineering, National Taipei University of Technology, Taipei, 10608, Taiwan; c Department of Energy and Process Engineering, Norwegian University of Science and Technology, 7491, Trondheim, Norway; d Department of Mechanical Engineering, University of Engineering and Technology, 39161, Lahore, Pakistan; e Department of Biosystems, KU Leuven, Leuven, 3001, Belgium

**Keywords:** Human thermal comfort, Tractor cabin, Design and development, Air-conditioning, CFD simulation

## Abstract

Tractors are manufactured without air-conditioned cabins in Pakistan. This leads to thermal discomfort for tractor operators working under direct solar exposure. Therefore, this study aimed to design and install an air-conditioned cabin on a tractor. Experiments were undertaken to evaluate the installed cabin performance under two scenarios i.e., conventional (S–I) and enhanced (S-II) air distribution. Computational fluid dynamics (CFD) simulations were used to analyze airflow and calculate thermal comfort indices. The results showed that the air-conditioned cabin attained optimum thermal conditions under the enhanced air distribution scenario (S-II). In this scenario, the inside cabin temperature was an average of 27.4 °C, compared with 30.4 °C in S–I. The relative humidity remained similar in both scenarios, around 53 %. The temperature difference between the cabin and the ambient environment was 11.09 °C in S-II, aligning with the thermal comfort conditions outlined in ISO 14269–2. Furthermore, the CFD simulations showed a predicted mean vote (PMV) index of 0.61 and the percentage people dissatisfied (PPD) index of 26.5 %. These results also confirm the provision of optimum thermal conditions for operator inside the cabin. The simulations also demonstrated good agreement with experimental data, with a small difference in air temperature (2 °C) and relative humidity (5.8 %). In the light of these findings, this study recommends installation of air-conditioned cabin on tractors with enhanced air distribution (S-II) in Pakistan to improve thermal comfort of operators.

## 1 Introduction

Human thermal comfort is state of mind which expresses satisfaction with surrounding thermal environment [1]. Being a function of different psychological and physiological attributes of a person, it is difficult to maintain human thermal comfort in indoor environments [2,3]. It is more complicated in vehicles, where several complexities occur due to transient microclimate [4,5] and direct solar exposure in the case of tractors and other agricultural machinery [[6], [7], [8]]. Several personal and environmental factors are involved in providing optimum thermal comfort for tractor operator [[9], [10], [11], [12]]. To overcome the impact of these factors, air-conditioned cabins are installed on tractors and other agricultural machinery to maintain optimum thermal comfort. Lack of optimum thermal comfort can adversely impact the ability of the driver to focus and stay concentrated while driving [[13], [14], [15]]. Thus, proper designing of tractor cabin and effectiveness of air-conditioning system are crucial determinants to ensure thermally comfortable environment [[16], [17], [18]]. In this regard, different types of air-conditioning systems have been proposed in the existing literature for agricultural applications [[19], [20], [21]].

International Organization for Standardization (ISO) has developed standards on design considerations for air-conditioning systems in tractor and other agricultural machinery. In this regard, the standard AS–ISO–3411 [22] provides a detailed guide for the design of tractor cabins. This standard briefly explains the cabin envelope and preferred space considerations for the operator. In contrast, ISO 14269–2 [23] discusses the procedural norms for the performance evaluation of air-conditioning system within tractor cabins and other self-propelled agricultural machinery. The ISO 14269-2 discusses two distinctive methods to assess thermally agreeable conditions within the tractor cabins, founded on the principles of thermal comfort zone and temperature difference between ambient and indoor environments. However, ISO-7730 introduces a more widely accepted methodology for evaluating optimal thermal comfort conditions based on predicted mean vote (PMV) and percentage people dissatisfied (PPD) [24,25]. PMV is an index on the thermal sensation scale ranging from +3 to −3 representing too hot - too cold thermal sensations, respectively [26]. PPD is the percentage of people dissatisfied with a specific PMV. Other similar standards for vehicular and agricultural machines air-conditioning applications have also been developed by ISO [27,28]. The adherence to standardization facilitates the development of efficient air-conditioning cabin for tractors and other self-propelled agricultural machinery. Several studies have investigated the performance of air-conditioning cabin for tractors, adhering to the aforementioned standards. Kaufman et al. [29] investigated the thermal comfort inside 16 tractor cabins, concluding that PMV index is a better indicator of thermal comfort than cabin temperature. Hwang and Kim [30] evaluated the thermal comfort in 31 tractor cabins based on the PMV index, revealing that majority of cabins fell outside the thermal comfort zone, with only 25 % of the tested tractors achieving PMV index within +0.5 to −0.5.

In a different vein, Oh et al. [16] conducted an experimental study to assess proper vent location inside the tractor cabin, suggesting that dashboard is the most suitable location for vents. However, Kabeel et al. [10] and Hou et al. [31] preferred ceiling for air-conditioner (AC) vents. Ruzic et al. conducted several experimental and numerical studies on tractor cabin air-conditioning and air flow analysis while considering operator thermal comfort [[32], [33], [34]].

Pakistan, being agricultural country, predominantly relies on tractors to perform various farm operations. It is pertinent to mention that all the tractors produced in Pakistan (about 0.7 million units to date) [35] lack air-conditioned cabins [36,37]. Remarkably, none of studies [16,29,30,32] have discussed the design and installation of an air-conditioned cabin on tractor. Therefore, in this study, an air-conditioned cabin was designed and installed on already manufactured tractor to provide thermal comfort to the operator. The performance evaluation of developed system was carried out through experimentation and simulation under specific climatic conditions characterizing Pakistan.

## 2 Methodology

### 2.1 Tractor cabin

**Fig. 1:**
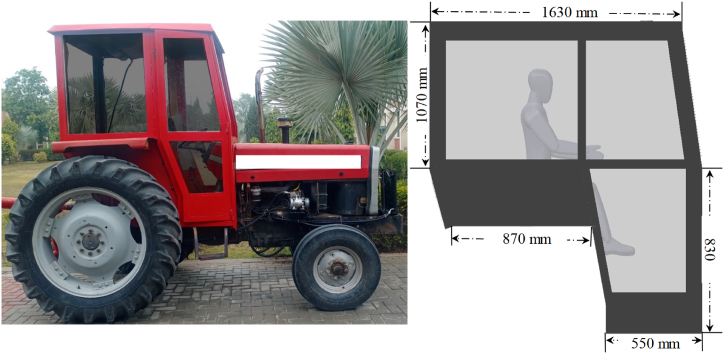
Pictorial representation of the developed tractor cabin and air-conditioning system.

A tractor cabin was designed in accordance with the specifications provided in the standard AS–ISO–3411 [22]. The developed cabin was installed on locally manufactured tractor in Pakistan, as shown in Fig. 1. The frame of the cabin was develpoed of cast iron while the walls were made of acryclic plastic. The metallic body of the frame was insulated with layers of insulation materials, i.e., carpet and rexine. The size of air-conditioning (AC) system was estimated based on different types of heat loads in the cabin. These constitute heat load inside the cabin due to the temperature difference (*Q*_*c*_), heat transfer through the cab roof (*Q*_*s,roof*_) and walls (*Q*_*s,wall*_) caused by solar radiation, heat gain due to air infiltration, heat gain from the engine powertrain (*Q*_*pt*_), and sensible (*Q*_*s*_) and latent (*Q*_*l*_) heat released by the operator [38]. On the other hand, heat rejection sources include solar heat reflected to the air (*Q*_*ref*_), heat rejection due to convection (*Q*_*con*_), and heat removed by the air-conditioning system (*Q*_*AC*_). Air-conditioning system removes the heat produced by the sources i.e., *Q*_*c*_, *Q*_*s,roof*_, *Q*_*s,wall*_, *Q*_*pt*_, *Q*_*s*_, and *Q*_*l*_. Therefore, heat rejection by air-conditioning system was considered in this study. However, heat rejection in terms of solar heat reflected to the air (*Q*_*ref*_) and heat rejection due to convection (*Q*_*con*_) were not considered in this study. Such exclusion of heat rejections are also described in literature [39]. Moreover, heat gain due to air infiltration was not considered in this study. It is because of pressurization of tractor cabin under the testing conditions [39]. The heat transfer between the ambient and the interior air due to the air temperature difference was calculated using Equation (1) [38].

(1) $$Q_{c\;}=UA\;\left(T_{o}-T_{cab}\;\right)$$

where, *Q*_*c*_ is the heat transfer through the cab envelope due to the temperature difference, *U is* total heat transfer coefficient of cab walls (W/m^2^K), *A is* surface area of cab walls (m^2^), *T*_*o*_ is ambient/outside air temperature (K) and *T*_*cab*_ is inside air temperature (K).

Similarly, heat transfer through the cab roof (*Q*_*s,roof*_) and walls (*Q*_*s,wall*_) caused by solar radiation can be calculated using Equation (2) and (3), respectively [39]. However, heat load from power train (*Q*_*pt*_) was calculated using Equation (4) [40]. Human thermal loads i.e., sensible and latent heat loads were determined using Equation (5) and ASHRAE guidelines, respectively [33,41].

(2) $$Q_{s,wall}=S_{i}.\tau +U\left(T_{i,G}-T_{cab}\right)$$

(3) $$Q_{s,roof}=U_{roof}\left(T_{i,roof}-T_{cab}\right)$$

(4) $$Q_{pt}={A_{floor}.U}_{floor}\left(T_{i,floor}-T_{cab}\right)$$

(5) $$Q_{o}=M_{h}.A$$

$$Q_{total}=Q_{c}+Q_{s,wall}+Q_{s,roof}+Q_{pt}+Q_{o}$$

where, *Q*_*s,wall*_ is the heat load through the cabin wall caused by solar radiation (W/m^2^), *S*_*i*_ is solar irradiance (W/m^2^), τ refers to solar transmitivity, *U* is heat transfer coefficient of cabin walls (W/m^2^K), *T*_*i,G*_ denotes temperature of the inner side of the cabin wall (K), *T*_*cab*_ represents inside air temperature (K), *Q*_*s,roof*_ is the heat load through the cabin roof caused by solar radiation (W/m^2^), *U*_*roof*_ refers to heat transfer coefficient of the cabin roof (W/m^2^K), *T*_*i,roof*_ denotes the inner side temperature of cabin roof (K), *Q*_*pt*_ is the heat load from powertrain (W/m^2^), *U*_*floor*_ represents heat transfer coefficient of the roof insulation material and taken as 5.56 W/m^2^K for carpet [42], *T*_*i,floor*_ is the temperature of floor (K) and taken as 50 °C in accordance with [39], *Q*_*o*_ is the sensible heat load from the operator (W), *M*_*h*_ is the metabolic heat released by the operator and was taken as 100 W/m^2^ for tractor's operator [43]. *A* refers to the surface area of the human body, which can be calculated by the formula given in Ref. [38]. Accordingly, overall heat loads were estimated about 3.42 kW for the ambient conditions of Multan. The evaporator of the recirculating type AC system has three varying speeds i.e., 5, 6.5, and 8.5 m/s with the maximum airflow rate of 195 m^3^/h. It was an internal ceiling evaporator with 6 vents installed above the operator's seat. The location of AC vents was specified based on the most suitable locations reported in the literature [10,31].

### 2.2 Experimentation

**Fig. 2:**
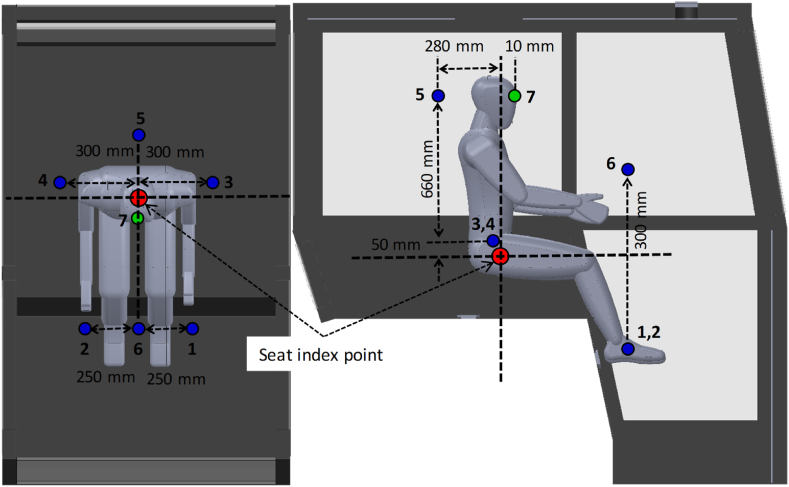
Spatial distribution and installation of air temperature, relative humidity, and velocity sensors inside the tractor cabin as described in ISO 14269–2 [23]. Points 1 to 6 represent the locations of temperature and relative humidity sensors while point 7 represents the air velocity measurement location.

Experiments were conducted during the summer season in Multan district, Pakistan (30.1575° N, 71.5249° E). Two air distribution scenarios were tested in this study i.e., air distribution without ducts referred as conventional air distribution scenario (S–I) and air distribution through ducts which is referred as enhanced air distribution scenario (S-II). In S–I, the cool air from the AC vents could freely flow through the cabin while in S-II, ducts were installed at two (out of six) AC vents to distribute air to the lower part of the cabin to tackle higher engine heat loads in this area. The diameter of ducts was equal to the diameter of AC vents i.e., 6.25 cm and the length of these ducts was 1.5 m. These ducts receive the air from AC vents and deliver it to the bottom of cabin i. e, near the operator's feet. The testing procedure was followed as described in the ISO 14269–2 [23]. Six sensors were installed inside the tractor cabin at the specified locations to record spatio-temporal variations in air temperature and relative humidity according to ISO 14269–2 [23]. The spatial distribution and locations of the installed sensors are shown in Fig. 2. Moreover, air velocity was also measured using an anemometer at the specified location. Specifications of the temperature and relative humidity sensors and anemometer are provided in Table 1. The data recorded by these sensors were used to evaluate the air-conditioning system performance. Uncertainty in measurement of temperature and relative humidity was calculated using Equation (6) [44]. Maximum uncertainty in temperature and relative humidity measurement were calculated as ±0.32 °C and ±5 %, respectively.

(6) $$\partial_{t\;}=\sqrt{\partial_{s}^{2}+\partial_{c}^{2}+\partial_{d}^{2}}$$

where, $\partial_{t}$ denotes total uncertainty, $\partial_{s}$, $\partial_{c}$, and $\partial_{d}$ represent uncertainties in sensors, calibration process, and data acquisition system. As the sensors do not have separate data acquisition units, $\partial_{d}$ was neglected while values for $\partial_{s}$ and $\partial_{c}$ were provided by the manufacturer [45].

**Table 1:** Specifications of the air temperature, relative humidity, and speed measuring instruments used in this study.

Model	Manufacturer	Parameter	Measuring range	Accuracy	Reference
GM1365	BENETECH	Temperature Relative humidity	−30 °C–80 °C 0–100 %	±0.3 °C ±5 %	[45]
GM1365	BENETECH				
UT363	UNI-T	Air speed	0–30 m/s	±(5%rdg+0.5)	[46]

The thermal comfort conditions were assessed based on the criteria defined in ISO 14269–2 i.e., (i) human thermal comfort zone, and (ii) temperature difference between ambient and inside cabin environments (ΔT) [23]. The human thermal comfort zone developed for tractors and other self-propelled agricultural machines on psychrometric chart represents temperature and relative humidity ranges between 24–27 °C and 15–85 %, respectively [23]. The second criterion of temperature differences described in ISO 14269-2 states that the air-conditioning system should be capable of reducing cabin temperature by a minimum of 11 °C compared with ambient temperature if ambient temperature is 38 °C or above [23]. The air thermal conditions inside the tractor cabin were tested against both the criteria, and human thermal comfort conditions were assumed to be achieved if either of the criteria was met. Moreover, thermal comfort indicators i.e., predicticted mean vote (PMV) and percentage people dissatisfied (PPD) were also calculated through airflow analysis.

### 2.3 Airflow analysis

Airflow analysis inside the tractor cabin was conducted using computational fluid dynamics (CFD) simulations in the *SOLIDWORKS* airflow simulation module [47]. The flow analysis was performed using k-ε turbulence model while the human thermal comfort parameters (i.e., predicted mean vote [PMV] and percentage people dissatisfied [PPD]) were estimated using the default human thermal comfort module of *SOLIDWORKS*. The basic continuity, momentum, and energy equations used in the simulation are Reynolds averaged Navier–Stokes equations. For steady-state conditions, these equations are written as Equations (7), (8), (9) [48]. However, the turbulence kinetic energy (*k*) and turbulence dissipation rate (ε) in k-ε turbulence model can be given by Equations (10), (11), respectively [8].

(7) ∇.(ρϑ)=0

(8) ∇.(ρϑ.ϑ)=∇.(τ)−∇P+ρg

(9) $$\nabla .\left(\vartheta .\left(P+\rho E\right)\right)=\nabla .\left[-\sum_{i}h_{i}j_{i}+k_{ef}\nabla T+\tau_{ef}.\vartheta \right]$$

where, Equations (7), (8), (9) are the continuity, momentum, and energy equations, respectively. The bold letters in the equations denotes vector quantities. ρ represents density (kg/m^3^), ϑ is the velocity (m/s), τ refers to stress (N/m^2^), P denotes static pressure (N/m^2^). Whereas g*,* E*,* $h_{i}$*,* $j_{i}$*,* $k_{ef}$*,* T*,* and $\tau_{ef}$ denote the gravitational force (N), energy per unit mass (J/kg), specific enthalphy (J/kg), diffucion flux (mol/m^2^s), thermal conductivity (W/m °C), temperature (°C), and effective stress (N/m^2^), respectively. Similarly, the k-ε turbulence model is given in the following equations [8].

(10) $$\nabla .\left(\rho k\vartheta \right)=S_{k}-\rho \epsilon +G_{k}-Y_{M}+G_{b}+\nabla .\left[\left\{\frac{\mu_{t}}{\sigma_{k}}+\mu \right\}\nabla k\right]$$

(11) $$\nabla .\left(\rho \epsilon \vartheta \right)=S_{\epsilon }-\rho C_{2}\frac{\epsilon^{2}}{\sqrt{v\epsilon }+k}+\rho C_{1}S_{\epsilon }+C_{3\epsilon }C_{1\epsilon }G_{b}\frac{\epsilon }{k}+\nabla .\left[\left\{\frac{\mu_{t}}{\sigma_{\epsilon }}+\mu \right\}\nabla \epsilon \right]$$

where, $S_{k}$ and $S_{\epsilon }$ are user defined terms (J/kg), $G_{k}$ is the turbulent kinetic energy related to the velocity gradient (J/kg), $Y_{M}$ refers to addition of fluctuations in turbulence (W/m^2^), $G_{b}$ is the turbulent kinetic energy related to buoyancy (J/kg), $\mu_{t}$ denotes turbulent viscosity (m^2^/s), $\sigma_{k}$ and $\sigma_{\epsilon }$ are turbulent Prandtl numbers for k and ϵ, respectively. Whereas $C_{1}$, $C_{2}$, $C_{1\epsilon }$, and $C_{3\epsilon }$ are the dimensionless constants. The values and details of all the constants and variables can be found in Ref. [16].

**Fig. 3:**
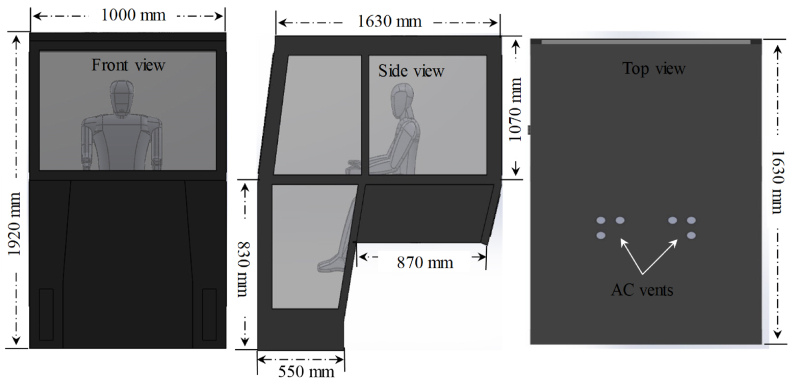
Design and dimensions of the actual tractor cabin used for airflow simulations.

**Fig. 4:**
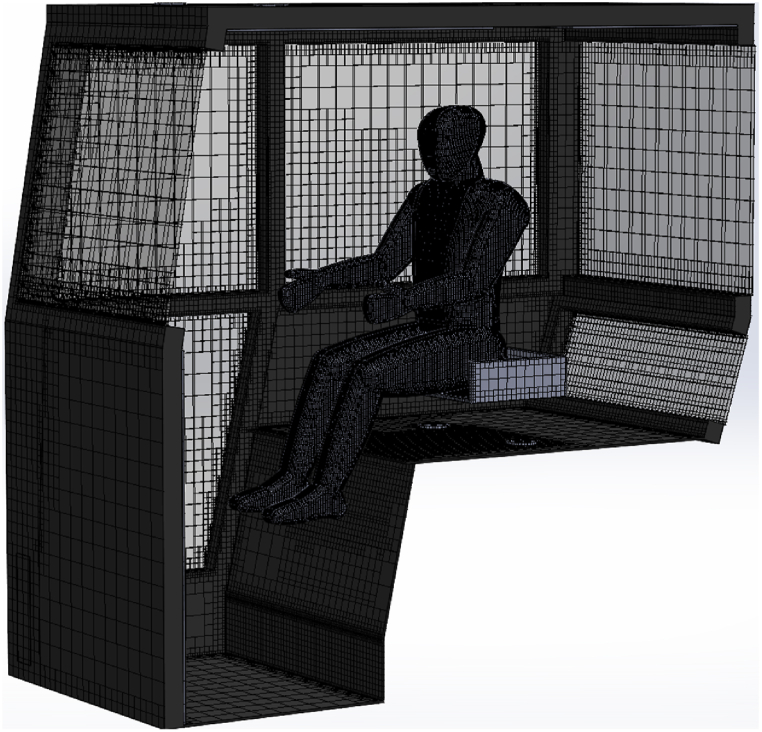
Development of meshing of the air-conditioned cabin for CFD simulations.

A symmetrical geometry of the actual tractor cabin and a mannequin (representing tractor operator) were designed in the software as shown in Fig. 3. Volume mesh of the tractor cabin was developed by default models to conduct CFD simulations as shown in Fig. 4. Mesh size was kept as 0.04 m in accordance to the previous literature [16]. Total solid and fluid cell in the cabin were 1,151,846 and 357,771, respectively while the level of solid and fluid cells refinement was taken as default i.e., 4. The air-conditioner vents were replicated by six air inflow sources of identical dimensions in the geometry and the location of these sources was specified as of actual location in the tractor cabin. The boundary conditions for airflow analysis were taken from experiments, actual environmental data, and calculations using governing equations. The air temperature, relative humidity, and velocity of inflow air from AC vents were recorded during experiments and used as boundary conditions in the simulation to replicate actual experimental conditions. Similarly, ambient air temperature, relative humidity, velocity, and solar radiation were also recorded during experiments and the thermal heat loads were calculated using Equations (1)–(5). Moreover, the cabin materials were specified from build-in materials library in the software. The thermal comfort indices (PMV and PPD) were calculated by activating the built-in “thermal comfort parameters” in the software. These indices can be calculated using Equations (12), (13), respectively [25].

(12) $$PMV=0.03e^{0.303}+0.028)\left\{\left(M_{h}-X\right)-3.05\left[5.73-0.007\left(M_{h}-X\right)-P_{a\;}\right]-0.42\left[\left(M_{h}-X\right)-58.15\right]-0.0173N\left(5.87-P_{a}\right)-0.0014M_{h}\left(34-T_{s}\right)-396.10^{-8}F_{c}\left[\left(F_{c}+273\right)-\;{\left(T_{m}+273\right)}^{4}\right]-F_{c}H_{c}\left(T_{c}-T_{s}\right)\right\}$$

(13) $$\text{PPD}=100-95\;\exp\;\left[-\left({0.03353\text{PMV}}^{4}+{0.2179\text{PMV}}^{4}\right)\right]$$

where, $M_{h}$, X, $P_{a\;}$, $T_{s}$, $F_{c}$, $T_{m}$, $H_{c}$, and $T_{c}$ refer to metabolic heat rate (W/m^2^), personal activity level (W/m^2^), vapor pressure (Pa), surrounding air temperature (°C), clothing factor (clo), mean radiant temperature (°C), heat transfer through convection (Wm^2^/°C), and temperature at clothing level (°C), respectively.

The simulation was performed under steady-state conditions therefore, the parameters changing with time were assumed to be zero [16]. However, other unknown parameters were calculated using governing equations and repeated simulations or taken from relevant literature. The metabolic heat rate ($M_{h}$) was taken as 100 W/m^2^ for tractor operator while personal activity level (X) was taken as 0 W/m^2^ because of negligible mechanical work by the operator, as described in Refs. [16,43]. The vapor pressure ($P_{a\;}$) was calculated through psychrometric calculations and surrounding air temperature ($T_{s}$) was taken as the average inside cabin air temperature during experiments. Moreover, clothing factor ($F_{c}$) was taken as 0.6 clo used for typical summer clothing as reported in the literature [16]. The remaining unknown parameters were calculated by the software through repeated simulations. Finally, the actual and simulated temperature and relative humidity were compared to check the validity of simulations.

This is the first benchmark study for air-conditioned tractor cabin in Pakistan. This study solves the local problem of unavailability of air-conditioned tractor cabin. Thereby, technical contributions of the study (for Pakistan) include the indigenization of cabin for existing tractors and performance evaluation under local thermal conditions. Moreover, the experiments were conducted under uncontrolled ambient environment. However, actual ambient thermal parameters like temperature and relative humidity during experiments were measured and used as boundary conditions in CFD simulations.

## 3 Results

**Fig. 5:**
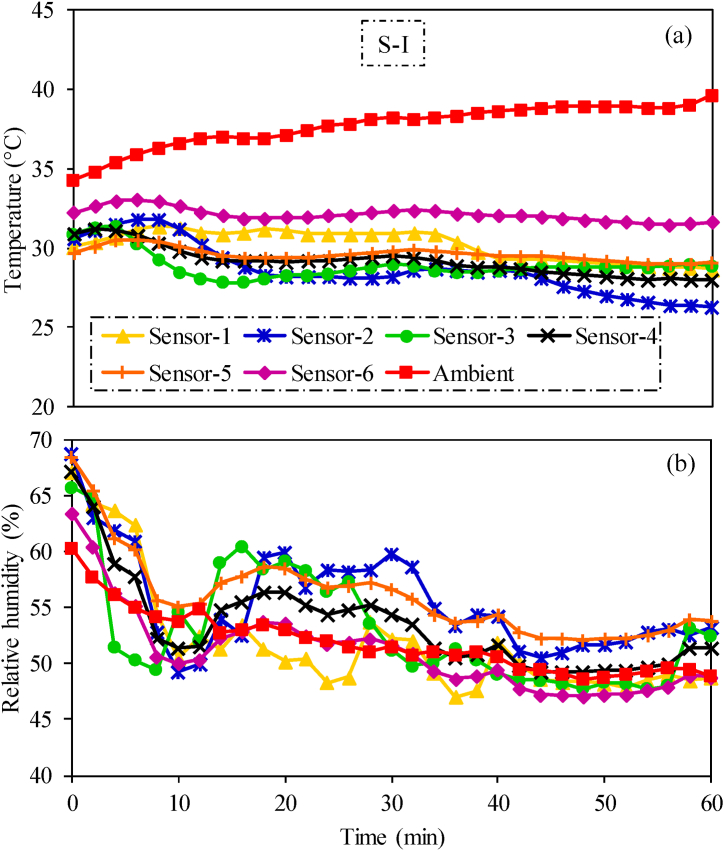
Experimental results of spatio-temporal variations in (a) temperature and (b) relative humidity at sensors 1–6 inside the tractor cabin in conventional air distribution (S–I) testing scenario.

Performance of the tractor air-conditioning cabin was first investigated for conventional air distribution scenario (S–I). Spatio-temporal variations in temperature and relative humidity inside the tractor cabin and ambient environment under S–I are shown in Fig. 5. The average ambient temperature and relative humidity during air-conditioning performance test were 38.3 °C and 51.9 %, respectively. However, the highest recorded temperature within the cabin reached 32 °C at sensor-6 during the test as shown in Fig. 5(a). The higher temperature at this location can be attributed to the heat loads from adjacent engine. This heat can be reduced by enhancing insulation between engine and cabin interior. On the other hand, the lowest temperature recorded, 28 °C, was at sensor-2. The highest spatial difference in temperature at different sensors was 4 °C which remains well within the maximum permissible spatial temperature difference of 5 °C, as specified in ISO-14269-2 [12]. Therefore, performance of the air-conditioning system can be evaluated under this experimental setup. The spatio-temporal variations in relative humidity inside the cabin shows a declining trend over time at all sensor locations relative to ambient conditions. The average highest and lowest relative humidity values were recorded as 56.06 % and 51.07 % at sensor-5 and sensor-6, respectively as shown in Fig. 5(b). The higher relative humidity at sensor-5 might be due to its location near to the operator's head. Higher latent heat load at this location impacted the relative humidity of the nearby air. On the other hand, lower relative humidity at sensor-6 is due to higher thermal loads from adjacent engine to this location. These results highlights the impact of latent and sensible heat sources on thermal comfort inside the cabin.

**Fig. 6:**
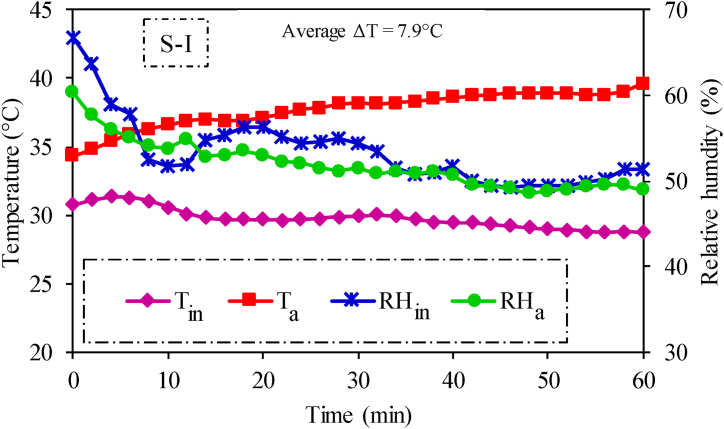
Temperature and relative humidity profiles inside the tractor cabin and ambient environment in conventional air distribution (S–I) testing scenario.

Temporal variations in average temperature and relative humidity inside the cabin and corresponding ambient conditions are presented in Fig. 6. Average inside cabin temperature and relative humidity were recorded as 30.4 °C and 53.5 %, respectively. The average temperature (30.4 °C) is higher than the thermal comfort zone (24–27 °C) specified in ISO 14269–2 [12]. However, the inside cabin temperature was found to 7.9 °C lower than ambient temperature. This also did not meet the minimum temperature difference criteria of 11 °C [12]. Therefore, the air-conditioning system could not achieve the minimum performance criteria of ISO 14269–2 under conventional air distribution (S–I) scenario. However, the cabin environment may still offer a more comfortable thermal experience for the operator in comparison to the ambient coditions.

**Fig. 7:**
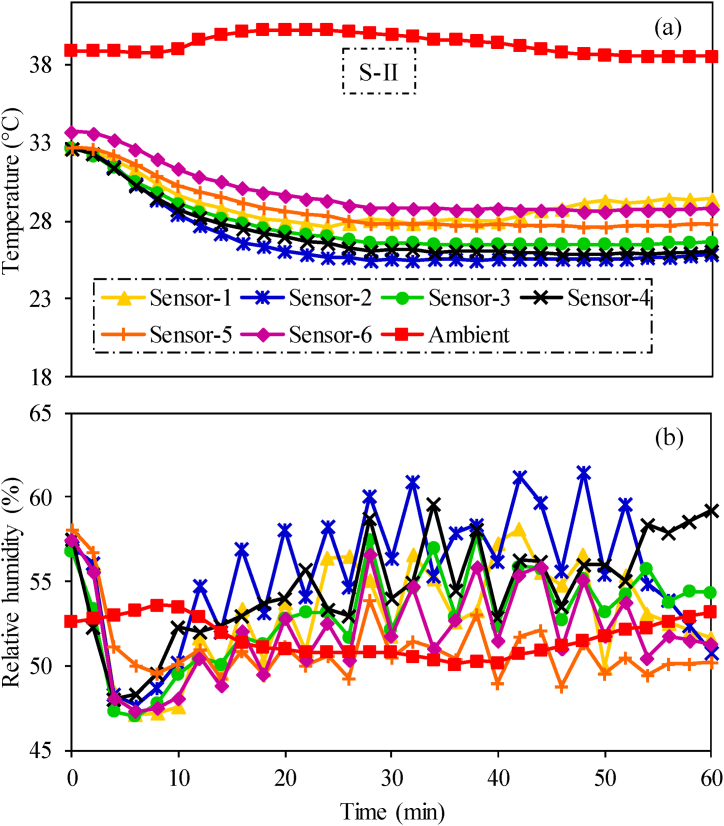
Experimental results of spatio-temporal variations in (a) temperature and (b) relative humidity inside the tractor cabin in enhanced air distribution (S-II) testing scenario.

Keeping in view the low performance of the cabin under S–I, a hypothesis was developed that proper distribution of AC air inside the cabin can enhance the performance of the system. Main basis of the hypothesis was the expected ability of AC air to tackle higher heat loads coming from the engine. Therefore, an air distribution scenario termed as enhanced air distribution (S-II) was introduced in the cabin. The spatio-temporal variations in temperature and relative humidity inside the cabin and ambient environment during S-II scenario are shown in Fig. 7. The average ambient temperature and relative humidity during the test were 39.3 °C and 51.7 %, respectively.

The temperature inside the cabin displayed variations across different sensor locations. Notably, the highest temperature (29.8 °C) was recorded at sensor-6 while the lowest (26.8 °C) was observed at sensor-2 as shown in Fig. 7(a). Significantly, the average temperature at sensor-2 location is within temperature limits (24–27 °C) of thermal comfort zone developed in ISO-14269-2 [12]. This is the only location where average temperature complied with the thermal comfort criteria. Average temperature at other sensor locations was slightly higher than 27 °C as shown in Fig. 7. However, at the end of the air-conditioning test, temperatures at sensor-3 and sensor-4 locations also converged to the thermal comfort temperature limits and recorded as 26.6 °C and 26.02 °C, respectively. These results show better air-conditioning performance of the cabin in S-II compared to S–I. However, there are notable temporal variations in relative humidity at specified sensors locations inside the cabin as shown in Fig. 7(b). These variations can be attributed due to latent heat loads and the periodic switching of the air conditioner after attainment of the desired temperature inside the cabin.

**Fig. 8:**
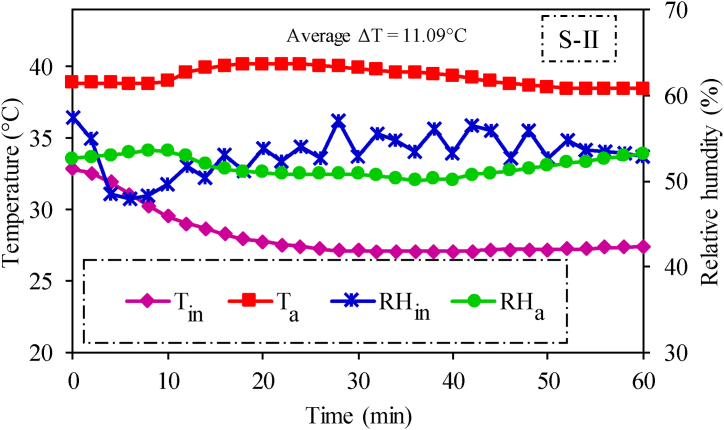
Temperature and relative humidity profiles inside the tractor cabin and ambient environment in enhanced air distribution (S-II) testing scenario.

Temporal variations in average temperature and relative humidity inside the cabin and ambient conditions are presented in Fig. 8. The average inside cabin temperature and relative humidity were recorded as 27.4 °C and 53.28 %, respectively. These results show that the average thermal conditions (temperature and relative humidity) inside the tractor cabin are very close to the first criterion of operator's thermal comfort as recommended by ISO 14269–2 [12].

Moreover, the second criterion (minimum difference between ambient and inside cabin temperature should be 11 °C) is clearly achieved as the temperature difference is 11.9 °C (Fig. 8). Thus developed hypothesis is true, and the air-conditioned cabin achieved thermal comfort conditions in enhanced air distribution scenario (S-II). Therefore, airflow analysis and calculation of PMV and PPD indices were further conducted by considering the boundary conditions of S-II.

**Fig. 9:**
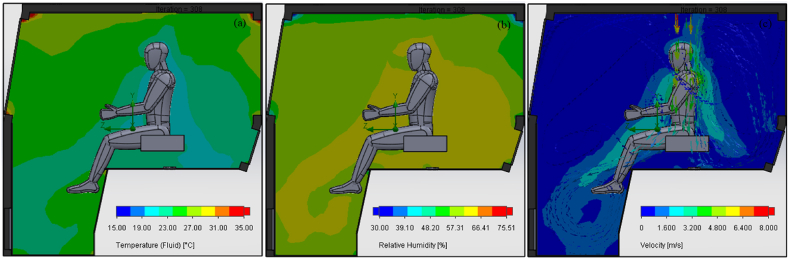
Cross sectional view of the tractor cabin showing CFD simulation results of air (a) temperature, (b) relative humidity, and (c) velocity.

CFD simulations were performed for airflow analysis to assess the inside cabin air temperature, relative humidity, velocity, PMV, and PPD indices. A cross sectional view of the simulated air temperature, relative humidity, and velocity inside the tractor cabin is shown in Fig. 9. The air temperature throughout the cabin varies between a range of 20–35 °C (Fig. 9(a)). However, it is lower in the near surroundings of the operator than cabin walls due to location of the AC vents. The overall temperature in operator's surroundings is within thermal comfort limits i.e., 24–27 °C [12]. Similarly, the corresponding relative humidity inside the tractor cabin is also within the thermal comfort zone (Fig. 9(b)). These results show that the simulated thermal environment inside the tractor cabin provides thermal comfort conditions to the operator in terms of air temperature and relative humidity. However, air velocity at the operator's head and chest is higher than the maximum allowable air velocity specified in ISO 14269–2 [12] (Fig. 9(c)). This discrepancy is due to the placement of the AC vents positioned above the operator's head. However, adjustment of air direction through the vents lever enables the reduction of air velocity up to acceptable limits in the actual tractor cabin.

**Fig. 10:**
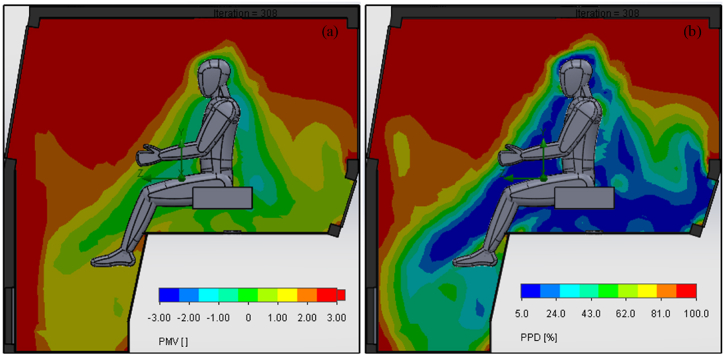
CFD simulation results of (a) predicted mean vote (PMV) and (b) percentage people dissatisfied (PPD) inside the tractor cabin.

Moreover, PMV and PPD indices are more relevant parameters related to human thermal comfort in conditioned spaces like tractor cabin [24]. The simulated results of these indices are shown in Fig. 10. As the thermal environment variates spatially inside the tractor cabin, the PMV and PPD values fluctuates accordingly. The PMV value near the operator's body lies around 0 (Fig. 10(a)) and the corresponding PPD value is less than 10 % (Fig. 10(b)). These results show that these indices are within thermal comfort limits and the operator is thermally comfortable inside the tractor cabin. Higher values of PMV and PPD near cabin walls are due to higher convective and conductive heat transfer from ambient environment, solar radiations, and engine heat. However, enhanced distribution of AC air in the bottom and engine side of the cabin reduces the impact of these heat loads, consequently establishing an environment that meets the criteria for thermal acceptability by the operator.

## 4 Discussion

**Fig. 11:**
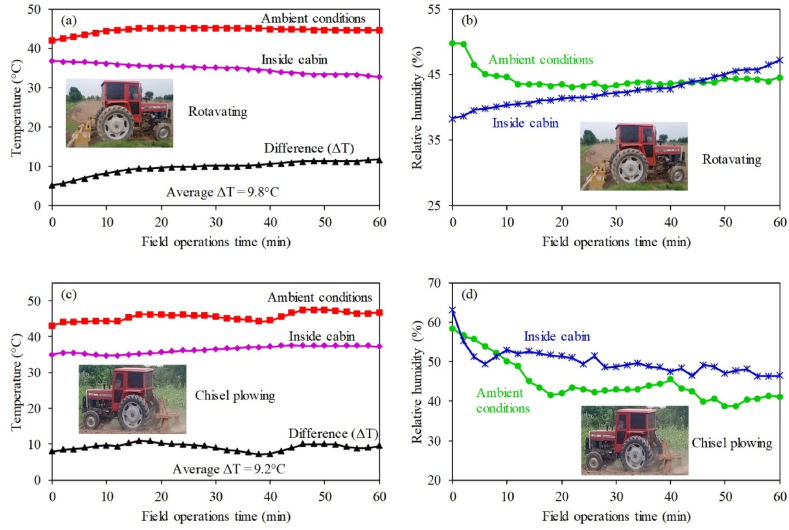
Temporal variations in ambient and inside cabin (a) temperature during rotavating, (b) relative humidity during rotavating, (c) temperature during chisel plowing, and (d) relative humidity during chisel plowing.

In conventional air distribution scenario (S-1), air from all the six AC vents moves downward from ceiling to reach operator's shoulders and chest. Afterwards, this air moves down to the feet of operator at sensor-2 location due to higher kinetic energy. Therefore, temperature at this location is the lowest as shown in results (Fig. 5). Similar results are reported in a study by Oh et al. [16]. The low temperature at this point is more prominent as the experiment progresses. It is due to continuous provision of cooled air from AC vents to this point. However, the highest was temperature was observed near operator's hands (sensor-6). It is due to the engine heat coming from dashboard near to this location. Due to this, the inside cabin environment could not achieve the temperature requirements of optimum thermal comfort zone as specified in ISO 14269–2 [23]. However, optimal thermal comfort zone for human varies in some other agricultural environments [19,49,50]. An air distribution scenario termed as enhanced air distribution (S-II) was introduced to increase air circulation inside the cabin. The air-conditioning performance of S-II was better than S-1 and clearly achieved the thermal comfort conditions. The performance of S-II system was further tested during field operations including rotavating and chisel plowing. The average temperature difference between ambient and inside cabin during rotavating and chisel plowing was 9.8 °C (Fig. 11(a)) and 9.2 °C (Fig. 11(c)), respectively. The operator felt thermal comfort condition even at temperature differenrece of 8.3 °C and 7 °C, as reported by Gupta et al. [51] and EI-Sheikha et al. [52], respectively. Similalry relative humidity during rotavating (Fig. 11(b) and chisel plowing (Fig. 11(d)) was also within thermal comfort limits [23]. Therefore, studied system also showed satisfactory performance under both field operations.

CFD simulations of S-II were conducted to further calculate PMV and PPD indices. A summary of the experimental and simulated results of air temperature, relative humidity, PMV, and PPD indices at different sensor locations inside the tractor cabin is provided in Table 2. Different studies provide comparison between simulated and experimental results for tracor cabin air-conditioning [16,[53], [54], [55]]. There are variations in simulated and experimental temperature and relative humidity at different sensor locations due to spatial differences and their distances from the AC vents. The average inside cabin air temperature was found as 27.4 °C (actual) and 25.9 °C (simulated) while corresponding average relative humidity values were 53.3 % and 59.0 %, respectively. These results show that the actual and simulated temperature and relative humidity inside the tractor cabin lie within the human thermal comfort zone specified in ISO 14269–2 [23].

**Table 2:** Air temperature, relative humidity, PMV, and PPD distribution at different sensor locations inside the tractor cabin for simulated air distribution scenario i.e., S-II.

Sensor location	Average experimental		Simulated		Difference		PMV	PPD (%)
	T (°C)	RH (%)	T (°C)	RH (%)	T (°C)	RH (%)		
Sensor 1	27.5	53.3	25.2	59.0	2.3	5.7	0.54	7.01
Sensor 2	26.2	55.9	25.0	62.3	1.2	6.4	0.12	5.00
Sensor 3	27.6	53.0	24.1	60.1	3.5	7.1	−0.24	5.08
Sensor 4	27.1	54.3	24.7	59.2	2.4	4.9	−0.17	5.02
Sensor 5	27.3	52.1	26.2	58.5	1.1	6.4	1.13	36.95
Sensor 6	28.8	51.0	30.2	55.0	1.4	4.0	2.3	99.92
Average	27.4	53.3	25.9	59.0	2.0	5.8	0.61	26.5

Similarly, the average PMV and PPD values inside the tractor cabin are 0.61 and 26.5 %, respectively. A PMV value of 0 indicates a thermally neutral state [56]. Values below 0 indicate a tendency for individuals to feel cool, while values above 0 indicate a tendency for individuals to feel warm. However, a slightly lower than 0 PMV is more comfortable to the operator inside cabin [57]. Therefore, the average PMV value inside the cabin shows a slightly warm sensation for some individuals. Moreover, the PPD value shows that 26.5 % of occupants may experience a degree of discomfort, primarily due to the slightly warm conditions inside the cabin. However, majority of people (73.5 %) are expected to experience thermal comfort. The average difference between experimental and simulated air temperature and relative humidity are 2.0 °C and 5.8 %, respectively in accordance with the previous study [16]. This differences between the actual and simulated values for temperature and relative humidity good agreement of simulation model with experimental data.

## 5 Conclusions

The present study aimed to install an air-conditioned cabin on a tractor and evaluate its performance during summer season in district Multan, Pakistan. In this regard, a tractor cabin (manufactured as per AS ISO 3411 design criteria) along with an air-conditioning system was installed on a 65 hp tractor. Experiments were conducted to test air-conditioning performance of the installed system under conventional (S–I) and enhanced (S-II) air distribution scenarios. Computational fluid dynamics (CFD) simulations were performed using experimental data of S-II as boundary conditions to conduct airflow analysis inside the tractor cabin. Air-conditioning performance of the cabin was evaluated based on its ability to provide thermal comfort to the operator. Therefore, thermal comfort was assessed based on three criterions i.e., (i) thermal comfort zone (ISO 14269–2), (2) air temperature difference (ISO 14269–2), and (3) PMV and PPD indices (ISO 7730). The results show that the average temperature difference between ambient and inside cabin environments was 7.9 °C during experiments under S–I, falling short of the recommended minimum differential of 11 °C. Moreover, the corresponding average inside cabin temperature (30.4 °C) and relative humidity (53.5 %) also did not meet the second criterion of thermal comfort i.e., human thermal comfort zone. Therefore, the installed cabin did not achieve optimum thermal comfort conditions under scenario S–I.

In S-II, the difference between ambient and inside cabin temperature was 11.09 °C, fulfilling the first criterion of thermal comfort. Moreover, the average inside cabin temperature (27.4 °C) and relative humidity (53.3 %) also nearly achieved the second criterion of human thermal comfort zone. Therefore, CFD simulations were performed for this scenario to conduct airflow analysis and calculate PMV and PPD indices. The average values of PMV and PPD indices inside the tractor cabin were 0.61 and 26.5 %, respectively. Moreover, the average difference between actual and simulated air temperature and relative humidity were 2.0 °C and 5.8 %, respectively, highlighting an acceptable level of agreement between experimental and simulated results. Based on the key findings of the study, it can be concluded that the installed cabin with enhanced air distribution as embodied in scenario S-II, was capable of providing thermal comfort to the tractor operator in Pakistan.

## Funding statement

This study receive no external funding for this research.

## Data availability statement

The data associated with this study has not been deposited into a publicly available repository. The datasets used and/or analyzed during the current study are available from the corresponding author upon request.

## CRediT authorship contribution statement

**Mahmood Riaz:** Writing – original draft, Visualization, Software, Methodology, Investigation, Formal analysis, Data curation, Conceptualization. **Muhammad Hamid Mahmood:** Writing – review & editing, Visualization, Validation, Supervision, Resources, Project administration, Methodology, Investigation, Data curation, Conceptualization. **Muhammad Nauman Ashraf:** Writing – review & editing, Visualization, Software, Methodology, Investigation, Formal analysis, Data curation. **Muhammad Sultan:** Writing – review & editing, Visualization, Validation, Supervision, Resources, Project administration, Methodology, Investigation, Conceptualization. **Uzair Sajjad:** Writing – review & editing, Visualization, Validation, Software, Investigation, Formal analysis. **Khalid Hamid:** Writing – review & editing, Visualization, Software, Methodology, Investigation, Funding acquisition, Formal analysis. **Muhammad Farooq:** Writing – review & editing, Visualization, Validation, Methodology, Investigation, Formal analysis. **Faming Wang:** Writing – review & editing, Visualization, Validation, Methodology, Investigation, Formal analysis.

## Declaration of generative AI and AI-assisted technologies in the writing process

No generative AI/AI-assisted technology is used in this paper.

## Declaration of competing interest

The authors declare that they have no known competing financial interests or personal relationships that could have appeared to influence the work reported in this paper.

## References

[1] ASHRAE Standard-55 (2010).

[2] Rupp R.F., Vásquez N.G., Lamberts R. (2015). A review of human thermal comfort in the built environment. Energy Build..

[3] Mishra A.K., Ramgopal M. (2013). Field studies on human thermal comfort — an overview. Build. Environ..

[4] Han T., Chen K.H. (2009). Assessment of various environmental thermal loads on passenger compartment soak and cool-down analyses. SAE Tech Pap.

[5] Xu J., Guo K., Sun P.Z.H. (2022). Driving performance under violations of traffic rules: novice vs. Experienced drivers. IEEE Trans Intell Veh.

[6] Kataev V., Markvo I., Khubiian K., Dimitrov V. (2021). Investigation of the reasons for the failures of the tractor microclimate system. E3S Web Conf..

[7] Mahmood M.H., Sultan M., Miyazaki T., Koyama S., Maisotsenko V.S. (2016). Overview of the Maisotsenko cycle – a way towards dew point evaporative cooling. Renew. Sustain. Energy Rev..

[8] Ashraf M.N., Mahmood M.H., Sultan M., Shamshiri R.R., Ibrahim S.M. (2021). Investigation of energy consumption and associated CO2 emissions for wheat–rice crop rotation farming. Energies.

[9] Simion M., Socaciu L., Unguresan P. (2016). Factors which influence the thermal comfort inside of vehicles. Energy Proc..

[10] Kabeel A.E., Sultan G.I., Zyada Z.A., El-Hadary M.I. (2010). Performance study of spot cooling of tractor cabinet. Energy.

[11] Shi J., Zhao B., He T., Tu L., Lu X., Xu H. (2023). Tribology and dynamic characteristics of textured journal-thrust coupled bearing considering thermal and pressure coupled effects. Tribol. Int..

[12] Xu J., Pan S., Sun P.Z.H., Hyeong Park S., Guo K. (2023). Human-Factors-in-Driving-Loop: driver identification and verification via a deep learning approach using psychological behavioral data. IEEE Trans. Intell. Transport. Syst..

[13] Norin F., Wyon D.P. (1992). Driver vigilance - the effects of compartment temperature. SAE Tech Pap.

[14] Alahmer A., Mayyas A., Mayyas A.A., Omar M.A., Shan D. (2011). Vehicular thermal comfort models; A comprehensive review. Appl. Therm. Eng..

[15] Xu J., Zhang X., Park S.H., Guo K. (2022). The alleviation of perceptual blindness during driving in urban areas guided by saccades recommendation. IEEE Trans. Intell. Transport. Syst..

[16] Oh J., Choi K., hee Son G., Park Y.J., Kang Y.S., Kim Y.J. (2020). Flow analysis inside tractor cabin for determining air conditioner vent location. Comput. Electron. Agric..

[17] Wu Z., Lin B., Fan J., Zhao J., Zhang Q., Li L. (2022). Effect of dielectric relaxation of epoxy resin on dielectric loss of medium-frequency transformer. IEEE Trans. Dielectr. Electr. Insul..

[18] Xu J., Park S.H., Zhang X., Hu J. (2022). The improvement of road driving safety guided by visual inattentional blindness. IEEE Trans. Intell. Transport. Syst..

[19] Mahmood M.H., Sultan M., Miyazaki T. (2022).

[20] Sultan M., El-Sharkawy, Miyazaki T., Saha B.B., Koyama S. (2015). An overview of solid desiccant dehumidification and air conditioning systems. Renew. Sustain. Energy Rev..

[21] Luo J., Zhao C., Chen Q., Li G. (2022). Using deep belief network to construct the agricultural information system based on Internet of Things. J. Supercomput..

[22] AS ISO 3411 (2020).

[23] ISO 14269-2 (2015).

[24] ISO 7730 (2015).

[25] Fanger P.O. (1970).

[26] Parsons K. (2020).

[27] ISO 14505-3 (2007).

[28] ISO 14505-4 (2021).

[29] Kaufman K R., Turnquist P K., Swanson R N. (1979). Thermal comfort in an air-conditioned tractor cab. Trans. ASAE (Am. Soc. Agric. Eng.).

[30] Hwang K.Y., Kim K.U., Kim K.U., Science B., Kim K.U., Science B. (2009). Evaluation of environmental comfort of tractor cabs. J Biosyst Eng.

[31] Hou X., Zhang X., Wang Y. (2023). Distribution of benzene and formaldehyde in tractor cabin: effects of components, ventilation conditions, and vent positions. Proc. Inst. Mech. Eng. - Part D J. Automob. Eng..

[32] Ružić D., Muzikravić V. (2005). Significance of tractor cab air-conditioning. Tractors Power Mach.

[33] Ružić D., Fakultet T.N., Sad N., Časnji F., tehničkih Nauka F. (2012). Analysis of tractor cab air distribution system efficiency by using CFD method. Tractors Power Mach.

[34] Ruzic D. (2012). Analysis of airflow direction on heat loss from operator’S body in an agricultural tractor cab. Aktual Zadaci Meh Poljopr.

[35] Government of Pakistan (2023).

[36] Millat Tractors Limited (2023). Agricultrual tractors. https://www.millat.com.pk/mtl-products/agricultural-tractors/.

[37] Al-Futtain Automotive (2023). Al-Ghazi Tractors Ltd. Pakistan.

[38] Ružić D.A. (2018). Airflow and thermal management in farm tractor cab. IOP Conf. Ser. Mater. Sci. Eng..

[39] Ruzic D., Casnji F. (2012). Thermal interaction between a human body and a vehicle cabin. Heat Transf. Phenom. Appl., InTech.

[40] Fayazbakhsh M.A., Bahrami M. (2013). Comprehensive modeling of vehicle air conditioning loads using heat balance method. SAE Tech. Pap..

[41] ASHRAE (1977).

[42] (2009). Thermal Insulation Performance of Carpet.

[43] (1994). ISO 8996.

[44] Uçkan İ., Yılmaz T., Hürdoğan E., Büyükalaca O. (2013). Experimental investigation of a novel configuration of desiccant based evaporative air conditioning system. Energy Convers. Manag..

[45] BENETECH. Temperature & Humidity Data Logger GM1365 n.d. http://www.benetechco.net/en/products/gm1365.html (accessed May 3, 2023)..

[46] UNI-T. UT363/UT363BT Mini Anemometers n.d. https://meters.uni-trend.com/product/ut363-ut363bt/#Specifications (accessed May 2, 2023)..

[47] Dassault Systèmes. Solidworks n.d. https://www.solidworks.com/(accessed May 3, 2023)..

[48] Anderson J.D., Wendt J. (1995).

[49] Ashraf M.N., Mahmood M.H., Sultan M., Khalid M., Miyazaki T. (2022).

[50] Mahmood M.H., Sultan M., Miyazaki T. (2020). Experimental evaluation of desiccant dehumidification and air-conditioning system for energy-efficient storage of dried fruits. Build. Serv. Eng. Technol..

[51] Gupta C.P., Abbas A., Bhutta M.S. (1995). Thermal comfort inside a tractor cab by evaporative cooling system. Trans Am Soc Agric Eng.

[52] El-Sheikha M.A., Abd-Alla H.E., El-Enany M.E. (2014). Development of environmental control techniques within enclosed tractor cab. J Soil Sci Agric Eng.

[53] Youssef Riachi D.C. (2014). A numerical model for simulating thermal comfort prediction in public transportation buses. Int. J. Environ. Protect. Pol..

[54] Ružić D. (2014). Numerical simulation of tractor operator thermal loads caused by solar radiation. Agric. Eng..

[55] Fujita A., Kanemaru J ichi, Nakagawa H., Ozeki Y. (2001). Numerical simulation method to predict the thermal environment inside a car cabin. JSAE Rev..

[56] Fiala D., Havenith G., Bröde P., Kampmann B., Jendritzky G. (2012). UTCI-Fiala multi-node model of human heat transfer and temperature regulation. Int. J. Biometeorol..

[57] Seo J.-W., Park J.-H., Choi Y.-H. (2012). Evaluation of thermal comfort and cooldown performance inside automotive cabin according to air-conditioning vent location. Trans Korean Soc Automot Eng.

